# Model based care in the ICU: A review of potential combined cardio-pulmonary models

**DOI:** 10.1371/journal.pone.0306925

**Published:** 2024-10-24

**Authors:** James Cushway, Liam Murphy, J. Geoffrey Chase, Geoffrey Shaw, Thomas Desaive, Cong Zhou

**Affiliations:** 1 GIGA-In Silico Medicine, University of Liège (ULg), Liège, Belgium; 2 Department of Mechanical Engineering, University of Canterbury, Christchurch, New Zealand; 3 Dept of Intensive Care, Christchurch Hospital, Christchurch, New Zealand; PLOS: Public Library of Science, UNITED KINGDOM OF GREAT BRITAIN AND NORTHERN IRELAND

## Abstract

Positive end-expiratory pressure results in a sustained positive intrathoracic pressure, which exerts pressure on intrathoracic vessels, resulting in cardiopulmonary interactions. This sustained positive intrathoracic pressure is known to decrease cardiac preload, and thus, decrease venous return, ultimately reducing both the stroke volume and stressed blood volume of the cardiovascular system. Currently, cardiovascular and pulmonary care are provided independently of one another. That positive end-expiratory pressure alters both stroke volume and stressed blood volume suggests both the pulmonary and cardiovascular state should be conjointly optimised. Optimising these systems in isolation may benefit one system, but have highly detrimental effects on the other. A combined cardiopulmonary model has the potential to provide a better understanding of patient specific pulmonary and cardiovascular state, as well as resulting cardiopulmonary interactions. This would enable simultaneous optimisation of all cardiovascular and pulmonary parameters. Cardiopulmonary interactions are highly patient specific and unpredictable, making accurate modelling of these interactions challenging. A previously validated cardiopulmonary model was found to have increasing errors at high positive end-expiratory pressures. A new iteration, the alpha model, was introduced to resolve this issue. This paper aims to review the alpha model against its predecessors, the previous cardiopulmonary model, and the original three chamber cardiovascular system model. All models are used to identify cardiovascular system parameters from measurements of 4 pigs during a preload reduction manoeuvre. Outputs and parameter estimations from models are compared to assess the relative performance of the alpha model against its predecessors. The novel alpha model was able to reduce model errors under high positive end-expiratory pressure, resulting in more accurate model outputs. At high positive end-expiratory pressures (20*cmH*_2_*O*), the alpha model had an average error of 11.24%, while the original cardiopulmonary model had a much higher error of 52.21%. Furthermore, identified outputs of the alpha model more closely matched those of the 3 chamber model than the previous cardiopulmonary model. On average, at high positive end-expiratory levels, identified model parameters from the alpha model showed a 6.21% difference to those of the 3 chamber model, while the cardiopulmonary model displayed a 39.43% difference. The alpha model proved to be more stable than the original cardiopulmonary model, making it a good candidate for model based care. However, it produced similar parameter outputs to the simpler three chamber cardiovascular model, bringing into question whether the additional complexity is justified, especially considering the low availability of clinical data in the ICU. There is a critical need for model based care to guide important procedures in ICU, such as fluid therapy. Candidate models should be continuously reviewed in order to guarantee the best possible care.

## Introduction

The respiratory system is known to cause cardiopulmonary interactions, occurring during the respiratory cycle. As the lungs inflate, they fill the thoracic cavity, pushing on the heart and intrathoracic blood vessels. Furthermore, positive pressure ventilation (PPV) and positive end-expiratory pressure (PEEP) result in a sustained positive intrathoracic pressure (ITP), exerting further forces on intrathoracic vessels. The resultant effects on the cardiovascular system (CVS) are highly complex and unpredictable, due to their highly patient-specific nature and complex mechanical etiology [1–6]. This unpredictable and patient-specific nature of cardiopulmonary interactions presents a difficult challenge for model-based cardiovascular monitoring and decision support in the ICU.

While model-based decision support is an emerging field [7], cardiovascular decision support for fluid administration to improve SBV and avoid excessive fluid administration is an important clinical area which has not been well-studied [8]. Excessive fluid administration also significantly impacts lung mechanics and function, and thus mechanical ventilation. The inter-relationship between cardiovascular system management and pulmonary system management is thus stronger, and both need to be accounted for in any decision support so their model-based management can be jointly achieved and optimised.

Initially, a simple 3 chamber model was presented as a potential candidate to provide model-based care in the ICU [7, 9]. However, this model excludes the pulmonary circulation and thus can not incorporate cardiopulmonary interactions. Therefore, a simple, clinically validated single chamber respiratory model [10–16] was coupled with the three chamber model, allowing cardiopulmonary interactions to be investigated [17, 18]. The coupling was achieved by subjecting the intrathoracic chambers of the 3 chamber model to ITP calculated from the respiratory model. However, the model suffered from increased model errors under high PEEP conditions due to exaggerated effects of ITP on the venous chamber. Recently, Cushway *et al* investigated improving this cardiopulmonary model [19] by adding a novel *α* parameter modulating applied ITP to intrathoracic chambers to reduce model errors.

This paper aims to assess the clinical relevance of the model and its novel parameter, and validate it against its predecessors, the 3 chamber model [7, 9] and the original cardiopulmonary model [17, 18].

## Methods

### Cardiopulmonary model

**Fig 1:**
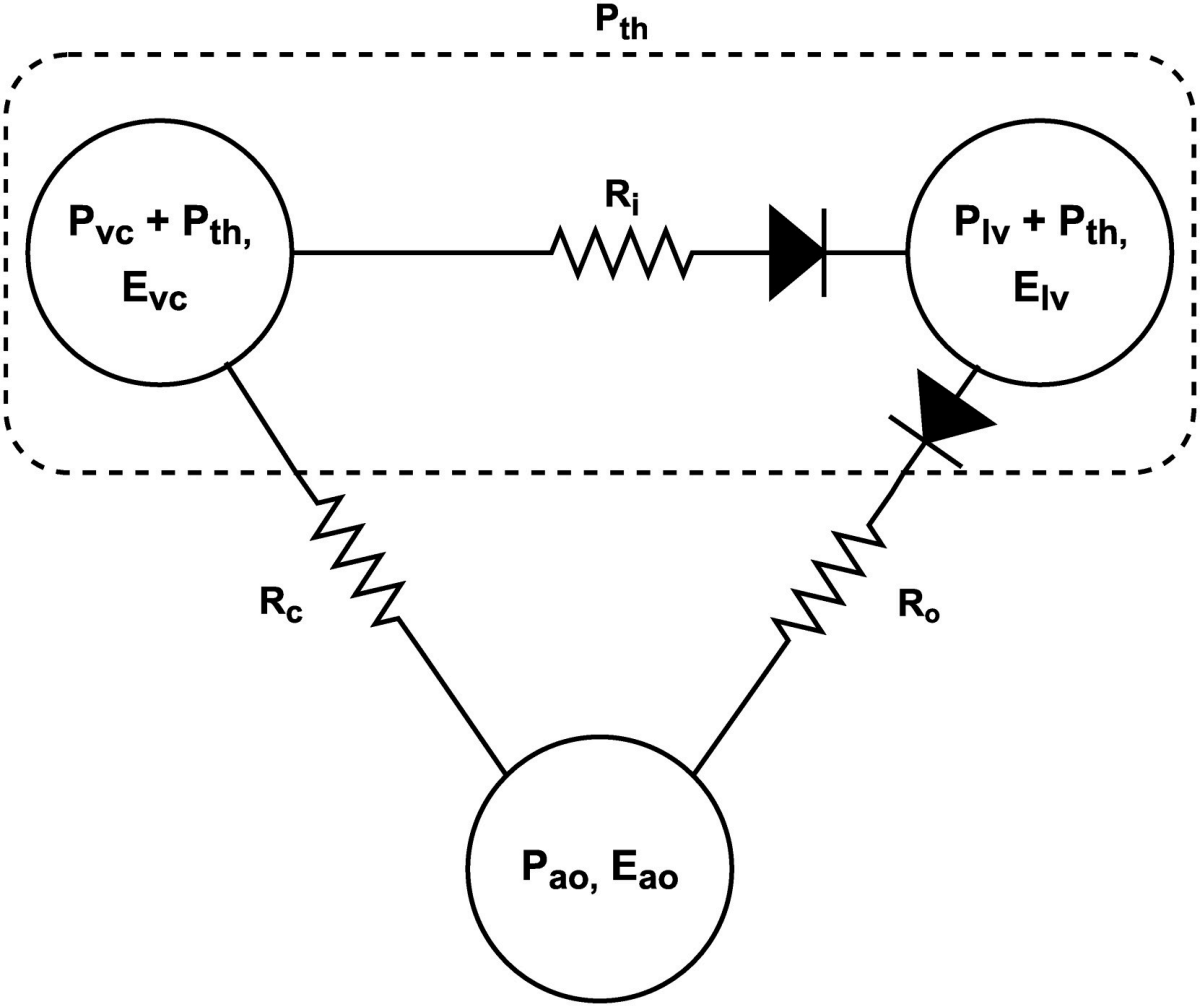
Coupled cardiopulmonary model. Coupled cardiopulmonary model with intra-thoracic pressure, *P*_*th*_, applied to the vena cava and left ventricle.

The cardiopulmonary model couples two previously clinically validated models, a 3 chamber cardiovascular model [7, 9, 17, 18], and a single chamber respiratory model [10–13]. The models are coupled through thoracic pressure, as seen in Fig 1.

During mechanical ventilation (MV), airway flow is determined using Ohm’s law:

$$\begin{array}{c} Q_{aw}\left(t\right)=\frac{P_{aw}\left(t\right)-P_{lung}\left(t\right)}{R_{aw}}, \end{array}$$ (1)

where *P*_*aw*_ is the airway pressure supplied by the ventilator, *P*_*lung*_ is the lung pressure and *R*_*aw*_ is the airway resistance. The lung is a passive chamber, and the lung pressure can thus be defined:

$$\begin{array}{c} P_{lung}\left(t\right)=E_{rs}V_{lung}\left(t\right)+PEEP \end{array}$$ (2)

where *V*_*lung*_ is the lung air volume and *E*_*rs*_ is the respiratory system elastance.

Respiratory system elastance is comprised of lung elastance and chest wall elastance:

$$\begin{array}{c} E_{rs}=E_{lung}+E_{wall} \end{array}$$ (3)

Lung volume is determined by airway flow and PEEP:

$$\begin{array}{c} V_{lung}\left(t\right)=\int Q_{aw}\left(t\right)dt+\frac{PEEP}{E_{rs}} \end{array}$$ (4)

where the integral of airway flow, ∫*Q*_*aw*_(*t*)*dt*, produces the tidal volume, *V*_*c*_(*t*).

Airway flow and pressure, and lung volume are provided by the ventilator. Substituting Eqs 2 into 1 yields airway pressure:

$$\begin{array}{c} P_{aw}\left(t\right)=R_{aw}Q_{aw}\left(t\right)+E_{rs}V_{lung}\left(t\right)+PEEP \end{array}$$ (5)

The two parameters of the system, *R*_*aw*_ and *E*_*rs*_, are identified via multiple linear regression of measured ventilator data.

ITP is directly proportional to lung volume and chest wall elastance:

$$\begin{array}{c} P_{th}\left(t\right)=E_{wall}V_{lung}\left(t\right) \end{array}$$ (6)

Substituting Eqs 4 into 6, thoracic pressure can be expressed:

$$\begin{array}{c} P_{th}\left(t\right)=E_{wall}V_{c}\left(t\right)+PEEP\frac{E_{wall}}{E_{rs}} \end{array}$$ (7)

The coupling between the respiratory and cardiovascular system models is achieved by subjecting intrathoracic chambers, namely the vena cava and left ventricle, to the ITP determined in Eq 7.

The aortic and vena cava chambers of the 3 chamber model are modelled as passive elastic chambers. Hence their pressures are dependent on their stressed volume and elastance:

$$\begin{array}{c} P_{ao}\left(t\right)=E_{ao}V_{s^{ao}} \end{array}$$ (8)

$$\begin{array}{c} P_{vc}\left(t\right)=E_{vc}V_{s^{vc}} \end{array}$$ (9)

Where *P*_*ao*_, *E*_*ao*_ and $V_{s^{ao}}$ represent aortic pressure, elastance and stressed volume respectively and *P*_*vc*_, *E*_*vc*_ and $V_{s^{vc}}$ represent vena cava pressure, elastance and stressed volume, respectively.

The left ventricle is an active chamber modelled using time varying elastance [20]:

$$\begin{array}{c} P_{lv}\left(t\right)=e\left(t\right)E_{lv}V_{s^{lv}} \end{array}$$ (10)

Where *e(t)* is the driver function creating time-variance. The driver function used in this work is taken from [21].

Thoracic pressure acts to decrease the transmural pressure of vessels and chambers located within the thoracic chamber. The stressed volume of a chamber is governed by its transmural pressure [3, 22, 23]. With applied ITP, vena cava and left ventricle pressures are re-defined:

$$\begin{array}{c} P_{vc}\left(t\right)-P_{th}\left(t\right)=E_{vc}V_{s^{vc}} \end{array}$$ (11)

$$\begin{array}{c} P_{lv}\left(t\right)-P_{th}\left(t\right)=e\left(t\right)E_{lv}V_{s^{lv}} \end{array}$$ (12)

where the thoracic pressure, *P*_*th*_(*t*), decreases the transmural pressure of the vessels.

The flow into each of the chambers is modelled using Ohm’s law. Systemic flow is accordingly defined:

$$\begin{array}{c} Q_{sys}\left(t\right)=\frac{P_{ao}\left(t\right)-P_{vc}\left(t\right)}{R_{sys}} \end{array}$$ (13)

The flows in and out of the heart are controlled by valves, and are thus represented using piecewise functions:

$$\begin{array}{c} Q_{o}\left(t\right)=\{\begin{array}{cc} \frac{P_{lv}\left(t\right)-P_{ao}\left(t\right)}{R_{o}}\; & P_{lv}\left(t\right)>P_{ao}\left(t\right)\\ 0 & \text{else} \end{array} \end{array}$$ (14)

$$\begin{array}{c} Q_{i}\left(t\right)=\{\begin{array}{cc} \frac{P_{vc}\left(t\right)-P_{lv}\left(t\right)}{R_{i}}\; & P_{vc}\left(t\right)>P_{lv}\left(t\right)\\ 0 & \text{else} \end{array} \end{array}$$ (15)

The rate of change of stressed volume for each chamber is given by the difference of input and output flow:

$$\begin{array}{c} {\dot{V}}_{s^{ao}}\left(t\right)=Q_{o}\left(t\right)-Q_{sys}\left(t\right) \end{array}$$ (16)

$$\begin{array}{c} {\dot{V}}_{s^{vc}}\left(t\right)=Q_{sys}\left(t\right)-Q_{i}\left(t\right) \end{array}$$ (17)

$$\begin{array}{c} {\dot{V}}_{s^{lv}}\left(t\right)=Q_{i}\left(t\right)-Q_{o}\left(t\right) \end{array}$$ (18)

The CVS is a closed system and its total rate of change of volume is zero, yielding:

$$\begin{array}{c} {\dot{V}}_{s^{lv}}\left(t\right)+{\dot{V}}_{s^{ao}}\left(t\right)+{\dot{V}}_{s^{vc}}\left(t\right)=0 \end{array}$$ (19)

Integrating Eq 19 gives the constant stressed blood volume for the system, $V_{s^{3}}$:

$$\begin{array}{c} V_{s^{3}}=V_{s^{lv}}+V_{s^{ao}}+V_{s^{vc}} \end{array}$$ (20)

### Alpha model

The original cardiopulmonary model suffered reliability issues under high PEEP conditions [17–19]. This issue was likely due to exaggerated ITP applied to the vena cava chamber, as the vena cava is modelled as being entirely intrathoracic, where, in reality, only a small portion of the venous blood reservoir lies in the thoracic chamber. From Eq 11, *P*_*vc*_ must exceed ITP (*P*_*th*_), or the equation would yield a negative value for stressed blood volume, which is not physiologically possible. The alpha model was consequently created by adding an *α* parameter to modulate the percentage of ITP applied to the vena cava and left ventricle to reduce these errors, yielding [19]:

$$\begin{array}{c} P_{vc}=E_{vc}V_{s^{vc}}\left(t\right)+\alpha P_{th}\left(t\right) \end{array}$$ (21)

$$\begin{array}{c} P_{lv}\left(t\right)=e\left(t\right)E_{lv}V_{s^{lv}}\left(t\right)+\alpha P_{th}\left(t\right) \end{array}$$ (22)

where *α* represents the fraction of thoracic pressure transmitted to the vena cava and left ventricle. However, the value of *α* has only limited direct physiological relevance, even if model errors are reduced.

The final alpha model includes 10 cardiopulmonary parameters from the CVS and respiratory system models, as well as *α* for adjusting applied ITP. The final parameter vector is summarised in Table 1.

**Table 1:** Parameter summary.

Parameter	Parameter Description
*E*_*a*_	Aortic elastance
*E*_*lv*_	Left ventricle elastance
*E*_*vc*_	Vena cava elastance
*R*_*c*_	Systemic resistance
*R*_*i*_	Input resistance
*R*_*o*_	Output resistance
*V*_*s*,3_	Total stressed blood volume
*E*_*wall*_	Chest wall elastance
*α*	Parameter to modulate ITP

### Nominal parameters

Nominal parameter values are required for parameter identification. Where possible, parameters were identified using model equations and measurements. Where measurements were unavailable, values from the literature were used. A summary of the equations for each parameter is defined in Table 2.

**Table 2:** Parameter definitions.

Parameter	Parameter Definition
*E*_*a*_	$\frac{\Delta P_{a}\left(t\right)}{CO.T}$ [17]
*E*_*lv*_	$max_{T}\frac{P_{lv}\left(t\right)}{V_{lv}\left(t\right)}$ [7]
*E*_*vc*_	$\frac{\Delta P_{vc}\left(t\right)}{CO.T}$ [17]
*R*_*c*_	$\frac{{\bar{P}}_{a}\left(t\right)-{\bar{P}}_{vc}\left(t\right)}{CO}$ [7]
*R*_*i*_	$\frac{\int_{P_{vc}\left(t\right)>P_{lv}\left(t\right)}P_{vc}\left(t\right)-P_{lv}\left(t\right)dt}{SV}$ [7]
*R*_*o*_	$\frac{\int_{P_{lv}\left(t\right)>P_{ao}\left(t\right)}P_{lv}\left(t\right)-P_{ao}\left(t\right)dt}{SV}$ [7]
*V*_*s*,3_	${\bar{V}}_{lv}\left(t\right)+\frac{{\bar{P}}_{a}\left(t\right)}{E_{a}}+\frac{{\bar{P}}_{vc}\left(t\right)}{E_{vc}}$ [7]
*E*_*wall*_	$\frac{E_{rs}}{2}$ [24]

#### Parameter subset

To ensure practical identifiability [25, 26], a common subset of sensitive parameters was chosen for optimisation for all pigs. The subset used in this work is from [19]:

$$\begin{array}{c} P_{subset}=\left[E_{ao},E_{lv},R_{sys},SBV\right] \end{array}$$ (29)

### Output vector

The output vector used in this work is the same used from [19]:

$$\begin{array}{c} X_{output}=\left[{\bar{P}}_{ao}\left(t\right),\;\Delta P_{ao}\left(t\right),\;{\bar{P}}_{vc}\left(t\right),\;\Delta P_{vc}\left(t\right),\;{\bar{V}}_{lv}\left(t\right),\;SV,\Delta max\left(P_{ao}\left(t\right)\right)\right] \end{array}$$ (24)

where ${\bar{P}}_{ao}\left(t\right)$ and Δ*P*_*ao*_ are mean and pulse aortic pressure, ${\bar{P}}_{vc}\left(t\right)$ and Δ*P*_*vc*_ are mean and pulse vena cava pressure, ${\bar{V}}_{lv}\left(t\right)$ and SV are mean left ventricular volume and stroke volume, and Δ*max*(*P*_*ao*_(*t*)) is the maximum change of peak aortic pressure over a respiratory cycle.

#### Error function

The error function is a measure of accuracy for identified model outputs. If *y*^*ref*^ is a vector containing the *N*_*y*_ reference measurements and *y*(*p*) is the output vector of parameter vector, *p*, each output’s error is defined as the difference between the measurement and the output:

$$\begin{array}{c} e_{i}\left(p\right)=y_{i}^{ref}-y_{i}\left(p\right),\;i\in \left[1,N_{y}\right] \end{array}$$ (25)

The error function is then defined using the RMS of all errors:

$$\begin{array}{c} \psi \left(p\right)=\sqrt{\frac{\sum_{i=1}^{N_{y}}e_{i}{\left(p\right)}^{2}}{N_{y}}} \end{array}$$ (26)

### Experimental procedure

The experimental protocol was approved by the Ethics Committee for the use of animals at the University of Liege, Belgium between September—November, 2015 (Reference Number 14–1726). Eight male, pure Pietrain pigs weighing between 18.5 and 29 kg were sedated and anaesthetised by an initial intramuscular dose of Zoletil 100 (0.1 mL/kg) and Ketamine 1000 (0.1 mL/kg). Sedation and anaesthesia was maintained by a continuous infusion of Nimbex (1 mL/kg/h at 2 mg/mL), Sufenta (0.1 mL/kg/h at 0.005 mg/mL) and Thiobarbital (0.1 mL/kg/h) via a central venous catheter positioned within the superior vena cava. The pigs were mechanically ventilated (GE Engstrom CareStation) with a baseline positive end-expiratory pressure (PEEP) of 5 cmH_2_O and tidal volume of 270 mL. The heart was accessed via a median sternotomy, and an admittance pressure-volume catheter (Transonic, NY, USA) with a sampling rate of 250 Hz inserted into the left ventricle. Proximal aortic pressure was continually sampled using a pressure catheter (Transonic, NY, USA) with a sampling rate of 250 Hz. Euthanasia was performed via a bolus of Pentobarbital (30 mg/kg) and Sufentanil (5 *μ*g/kg) causing respiratory arrest. To ensure a diverse range of cardiac states was exhibited, several procedures were performed:

- A single infusion of endotoxin (lipopolysaccharide from E. Coli, 0.5 mg/kg injected over 30 minutes) to induce septic shock, which drives a change in afterload conditions and is associated with a large variety of effects including an inflammatory response and capillary leakage that may lead to hypovolemia, global tissue hypoxia and cardiac failure
- Several PEEP driven recruitment manoeuvres (both pre- and post- endotoxin infusion), which drive a change in preload conditions and are typically associated with a decrease in mean blood pressure and cardiac output
- One to four infusions of 500 mL saline solution over 30 minute periods (both pre- and postendotoxin infusion), simulating fluid resuscitation therapy, a key component of heamodynamic resuscitation in patients with severe sepsis, which results in a change in circulatory volume

The airway flow data for Pigs 1 and 5 gave no negative readings, rendering the data unusable for the cardio-pulmonary model. Pigs 2 and 3 both displayed unreliable data with multiple interruptions in the data stream and were thus also unusable for this study. The remaining pigs (Pigs 4, 6, 7 and 8) all had usable data and were used in this work. For ease of use, the pigs are respectively numbered Pigs 1, 2, 3 and 4 for this work.

#### Parameter identification

The parameter identification in this work was performed using MATLAB’s (The Mathworks, Natick, MA, USA) *fmincon* function. The parameter subset and error function were passed as parameters. Parameter estimation was performed for all 4 pigs over the first and last 5 beats for each PEEP level of the RM.

To find *α*, the parameter identification process was repeated for *α* ∈ {0, 0.01…1.0}. The value for *α* resulting in the lowest error was selected for each pig. This sensitivity analysis and parametric optimisation over *α* seeks to find the appropriate fraction of ITP transmitted and assesses how pig specific it is.

### Analysis

As mentioned previously, *α* accommodates for the exaggerated ITP applied to the entire venous blood supply. However, it is possible *α* also compensates for several other cardiopulmonary mechanisms occurring simultaneously to those caused by ITP. To identify the physiological relationships of *α*, optimal *α* was correlated with various physiological measures, specifically SV, functional residual capacity of the lungs (*V*_*frc*_), preload and afterload, where end-diastolic left ventricle volume (*V*_*lv*,*d*_) is used as a measure for preload, and output resistance (*R*_*out*_). Left ventricle end diastolic volume was taken from direct invasive measurements, and output resistance was calculated using the nominal parameter estimation shown in Table 2. Finally, *V*_*frc*_ is determined from respiratory data using a clinically validated lung model [14, 27, 28]

Furthermore, to fully assess model performance and reliability, identified parameters from all three models (three chamber, cardiopulmonary and alpha) were compared to assess how each model performed under changing PEEP conditions.

## Results

### Optimum alpha

The optimum *α* was identified as the value minimising model errors. The identified optimum *α* of each pig for each PEEP level is shown in Table 3.

**Table 3:** Optimum *α* of all pigs for all PEEP levels.

Pigs	0*cmH*_2_*O*	5*cmH*_2_*O*	10*cmH*_2_*O*	15*cmH*_2_*O*	20*cmH*_2_*O*
1	0.38	0.37	0.22	0.17	0.22
2	0.45	0.30	0.31	0.25	0.20
3	0.39	0.38	0.26	0.18	0.23
4	0.55	0.24	0.19	0.14	-

As PEEP increases, *α* tends to decrease to compensate for the increasingly exaggerated effect calculated ITP has on the system.

### Alpha relationships

**Fig 2:**
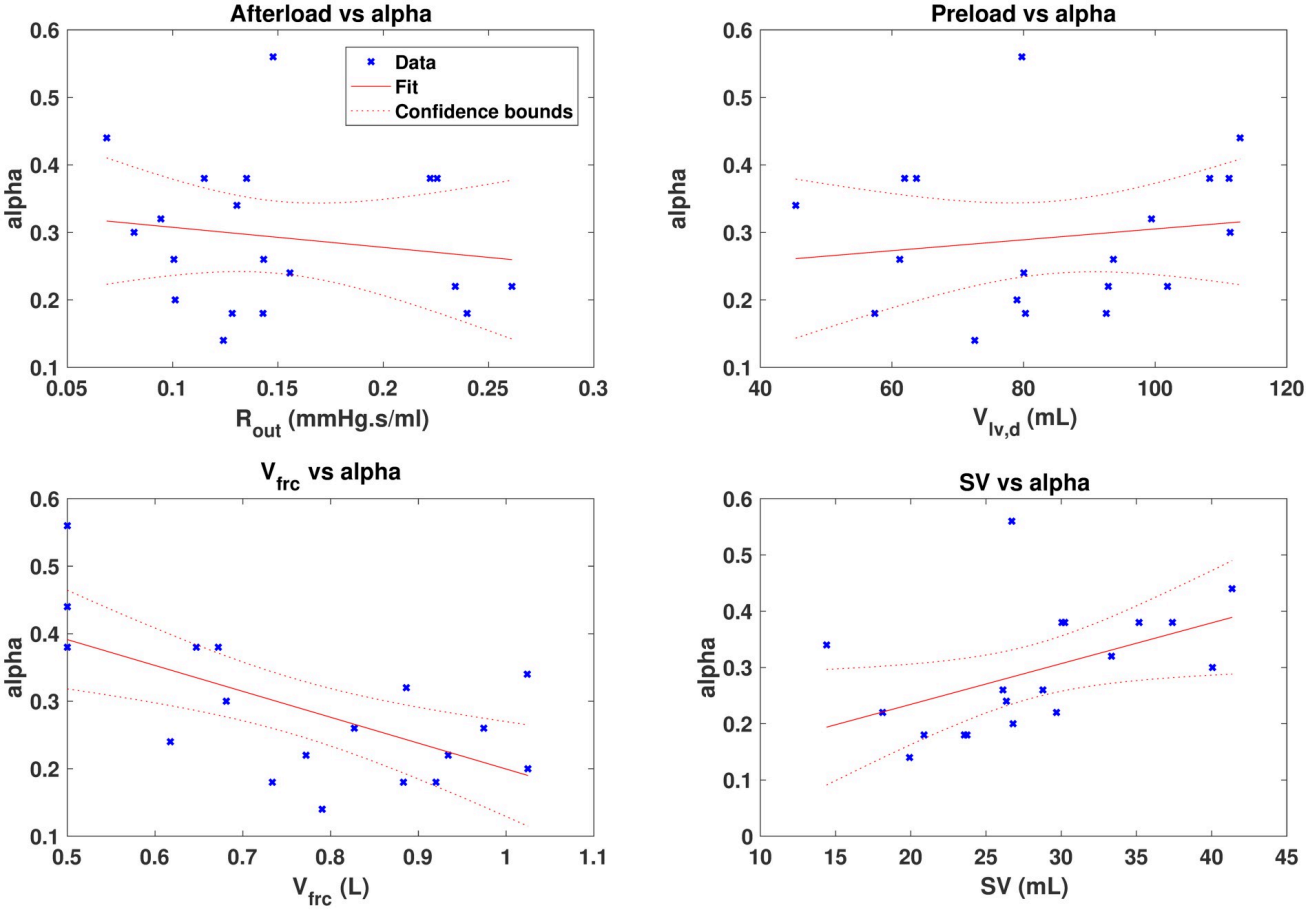
Physiological relationships of *α*. Physiological relationships of *α*. The top left shows afterload vs *α*, top right preload vs *α*, bottom left functional residual capacity of the lungs (*V*_*frc*_) vs *α* and bottom right stroke volume (SV) vs *α*.

Regression lines were fitted between *α* and a range of physiological measures indicative of known cardio-pulmonary interaction mechanics, including SV, *V*_*frc*_, preload (*V*_*lv*,*d*_) and afterload (*R*_*sys*_). The relationships are shown in Fig 2 and there associated *r*^2^ values are found in Table 4.

**Table 4:** *r*^2^ values for *α* relationships.

Parameter	*r* ^2^
Afterload	0.03
Preload	0.02
*V*_*frc*_	0.41
SV	0.24

The relationships between *α* and afterload, and *α* and preload, do not appear to be predictable, and as a result, produced very low *r*^2^ values of 0.03 and 0.02 respectively. However, the relationships between *α* and *V*_*frc*_, and *α* and SV, appear much stronger. *V*_*frc*_ and *α* show an inverse relationship, where *α* decreases as *V*_*frc*_ is increased, whereas *α* and SV show a direct relationship, where *α* increases with SV. Their respective *r*^2^ values were 0.41 and 0.24.

### Model comparisons

**Fig 3:**
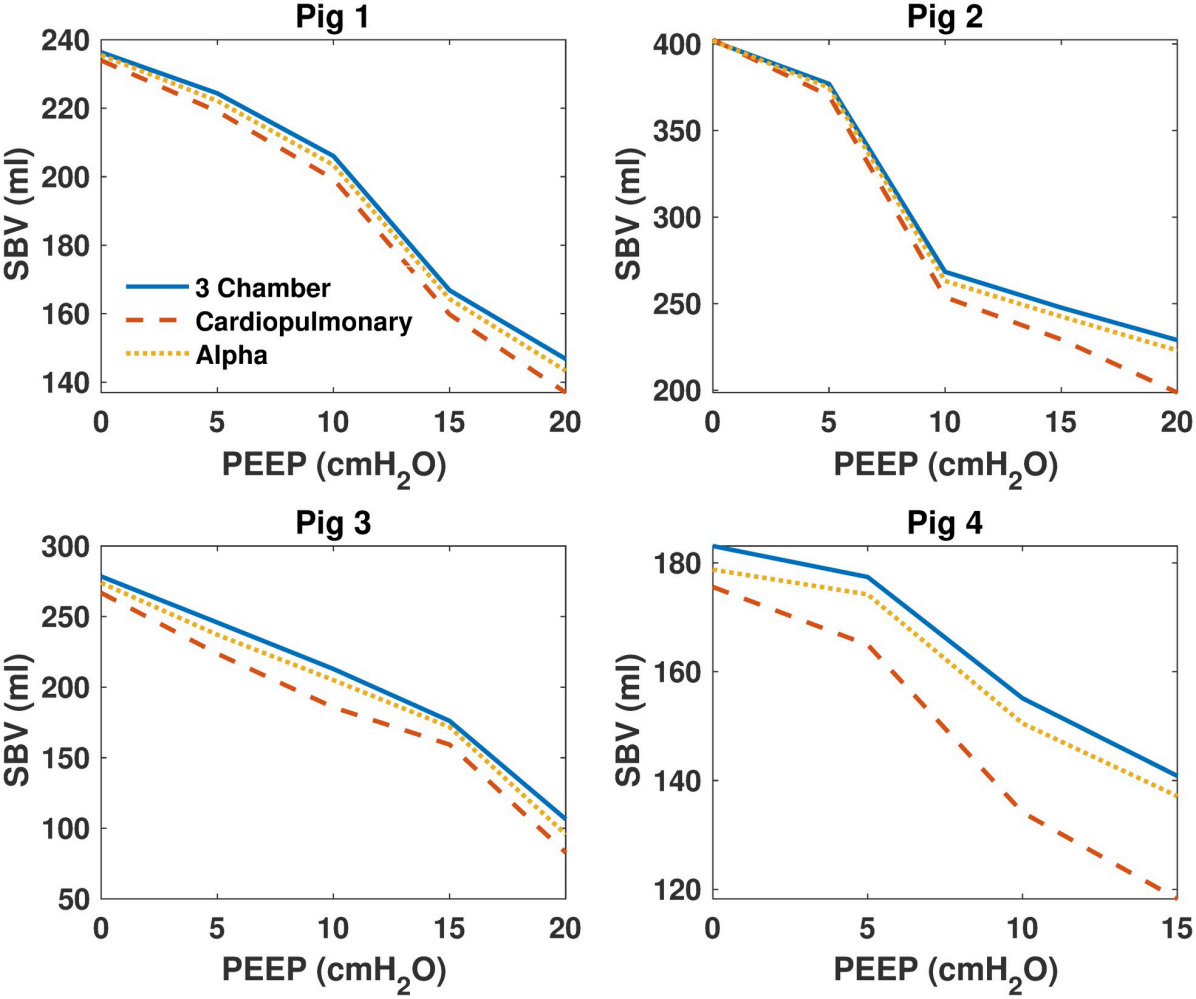
Identified stressed blood volume (SBV) of the 3 models for all pigs. Identified stressed blood volume (SBV) of the 3 models for all pigs. The 3 chamber model is shown in blue, the cardiopulmonary model in red and the alpha model in yellow.

**Fig 4:**
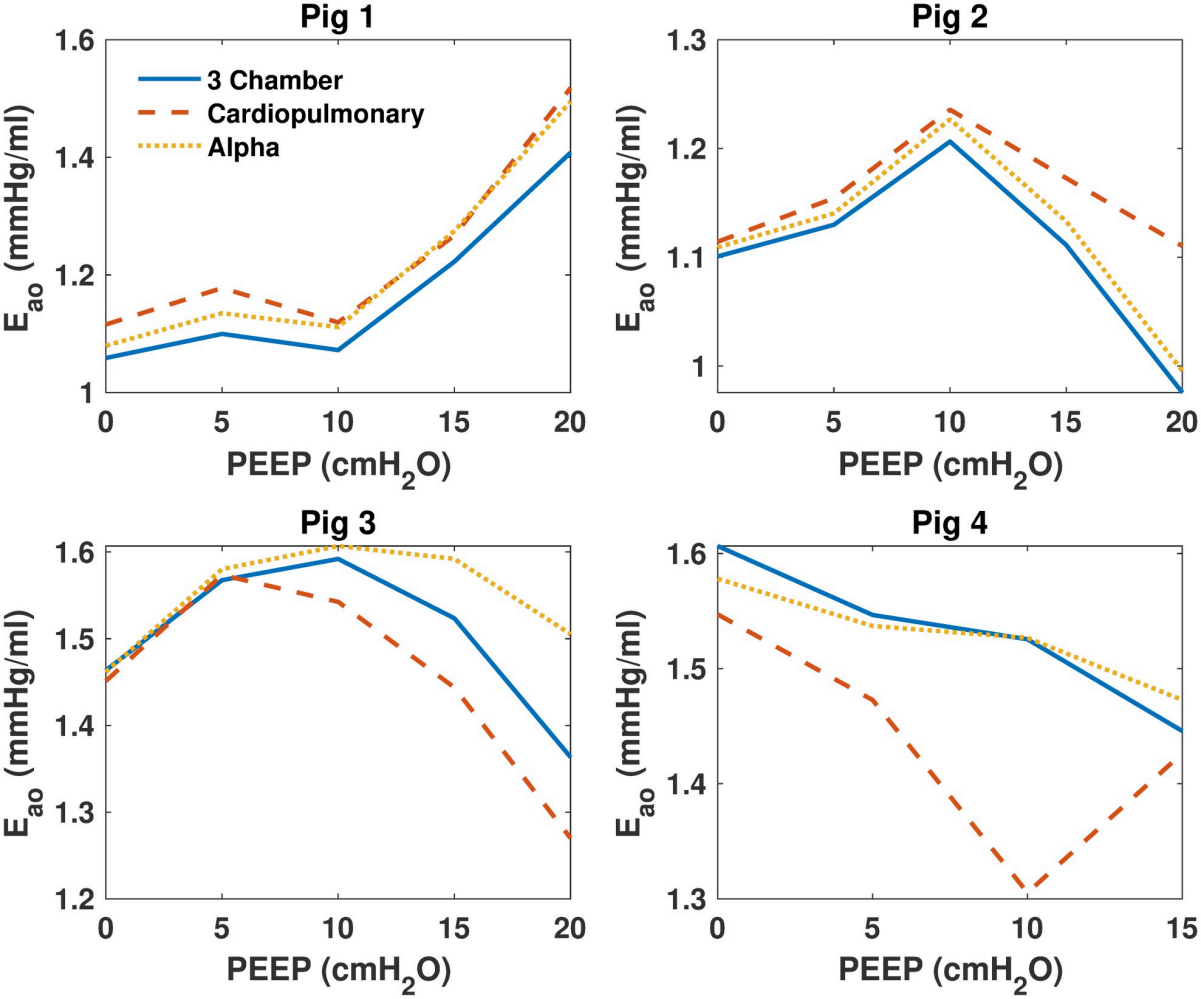
Identified aortic elastance (*E*_*ao*_) of the 3 models for all pigs. Identified aortic elastance (*E*_*ao*_) of the 3 models for all pigs. The 3 chamber model is shown in blue, the cardiopulmonary model in red and the alpha model in yellow.

**Fig 5:**
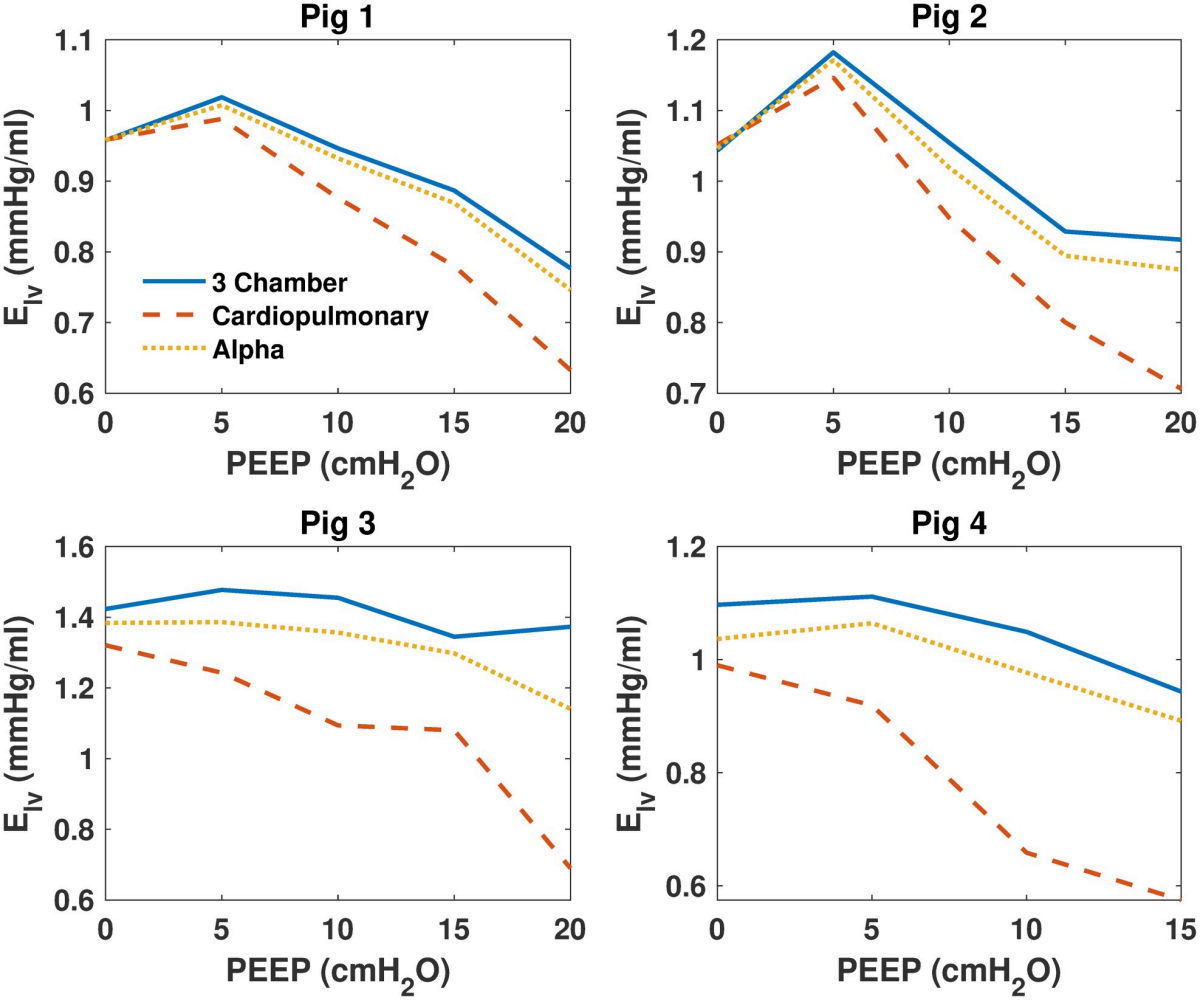
Identified left ventricle elastance (*E*_*lv*_) of the 3 models for all pigs. Identified left ventricle elastance (*E*_*lv*_) of the 3 models for all pigs. The 3 chamber model is shown in blue, the cardiopulmonary model in red and the alpha model in yellow.

**Fig 6:**
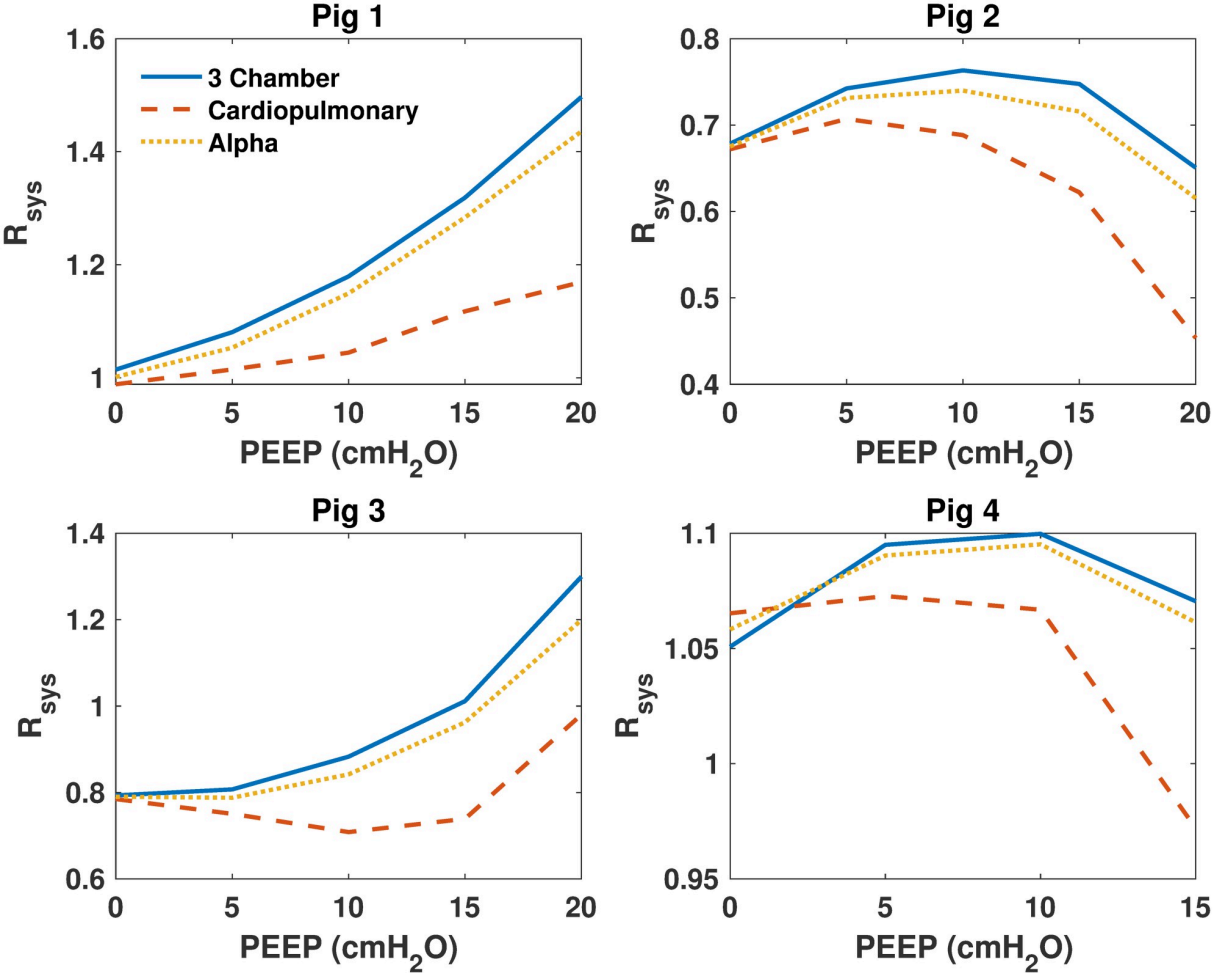
Identified systemic resistance (*R*_*sys*_) of the 3 models for all pigs. Identified systemic resistance (*R*_*sys*_) of the 3 models for all pigs. The 3 chamber model is shown in blue, the cardiopulmonary model in red and the alpha model in yellow.

Model parameters were identified for each model and then compared for all pigs over each PEEP step. Fig 3 shows identified SBV for all models, Fig 4 shows identified *E*_*ao*_ of all models, Fig 5 shows identified *E*_*lv*_ of all models. Finally, Fig 6 shows identified *R*_*sys*_ of all models.

**Fig 7:**
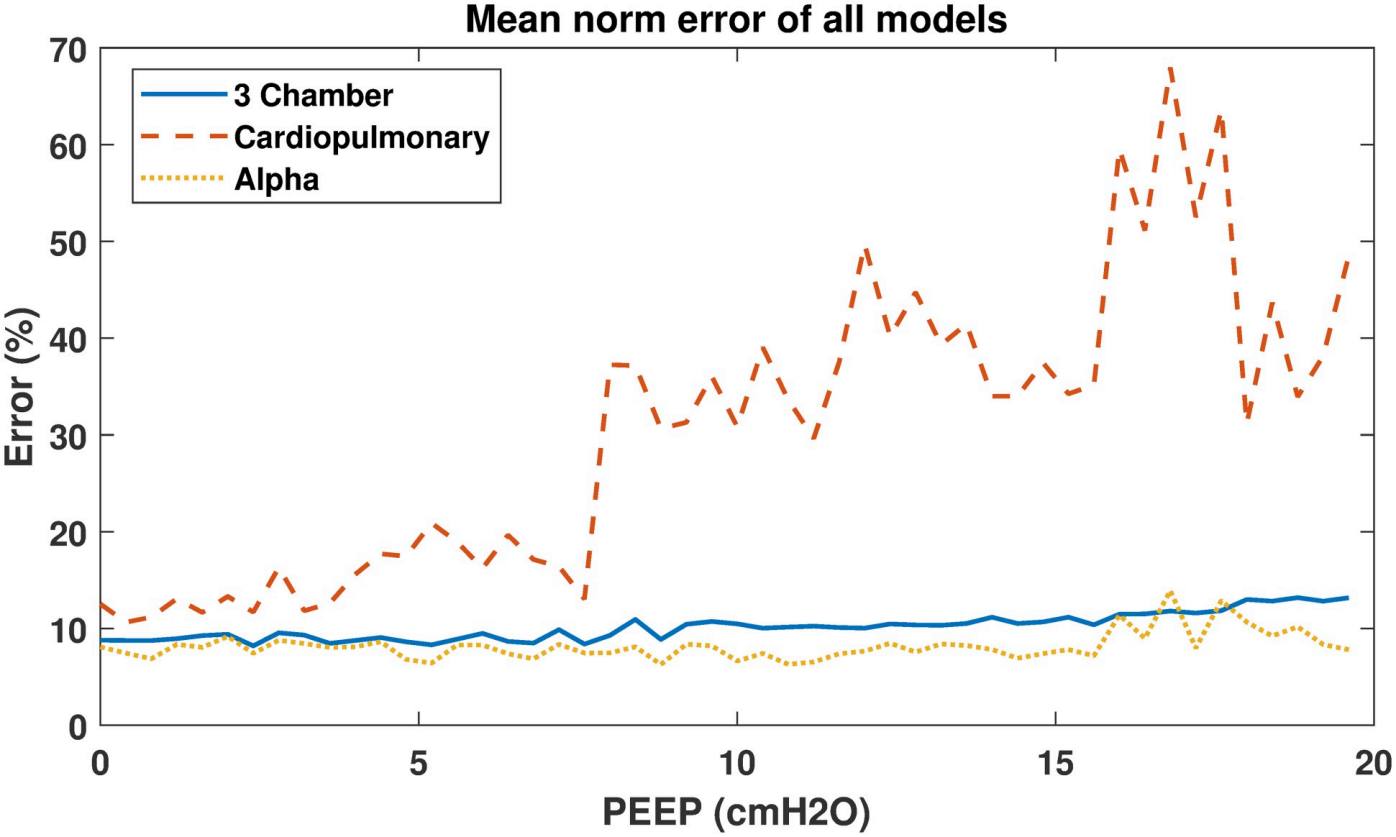
Average normalised error vs positive end-expiratory pressure (PEEP) of the 3 models. The average normalised error vs positive end-expiratory pressure (PEEP) of the 3 models. The 3 chamber model is shown in blue, the cardiopulmonary model in red and the alpha model in yellow.

Mean norm errors of each model were also compared to assess relative model performance. The normalised error of all pigs were averaged for each model and plotted, shown in Fig 7. The average normalised error per PEEP section can also be found in Table 5.

**Table 5:** Average error per positive end-expiratory pressure (PEEP) step for each model.

PEEP (*cmH*_2_*O*)	3 Chamber (%)	Cardiopulmonary (%)	Alpha (%)
0	8.74	12.61	7.95
5	9.12	18.51	7.76
10	10.47	35.40	7.43
15	10.78	42.69	7.66
20	11.83	52.21	11.34

## Discussion

### Effects of cardiopulmonary interactions

Before comparing relationships between *α* and various physiological mechanisms, it is important to understand the effect of cardiopulmonary interactions on the circulation, and how exactly they affect these physiological measures.

#### Preload

Positive pressure ventilation is associated with a decrease in left ventricular preload. However, left ventricular preload is not directly affected by PPV. Rather, the decrease in preload is a result of reduced SV from the right circulation due to multiple simultaneous mechanisms.

Firstly, PPV impedes venous return to the right ventricle. It is well documented that PEEP causes an increase in right atrial pressure (*P*_*ra*_) [1, 4, 6, 23]. Pressure of the upstream venous reservoirs, equal to mean systemic filling pressure (*P*_*msfp*_), is the upstream driving pressure for venous return, and *P*_*ra*_ is the downstream pressure. Thus, venous return is proportional to the pressure gradient between *P*_*msfp*_ and *P*_*ra*_ [24]. PPV is not known to cause significant changes to *P*_*msfp*_, and as a result, the increase in *P*_*ra*_ causes a decrease in venous return, reducing right ventricular preload.

Secondly, PPV also causes an increase in right ventricular afterload. Since the right circulation is located entirely within the thoracic cavity, increased ITP causes a decrease in transmural pressure of the vessels of the right circulation, causing a reduction of stressed volume of those vessels, and hence, a reduction in cross sectional area. The resistance of a vessel is inversely proportional to the fourth power of its diameter [24]. Thus, even small decreases of a vessel’s cross sectional area can greatly increase its resistance. Consequently, increases in ITP cause increases in pulmonary vascular resistance. Furthermore, increased *V*_*frc*_ due to PEEP compresses and stretches small vessels within the lungs, further increasing pulmonary vascular resistance [4, 6, 19, 23]. This increase in PVR through both ITP and *V*_*frc*_ causes an increase in right ventricular afterload.

The decrease of right ventricular preload, and increase in right ventricular afterload, causes a significant decrease in right ventricular SV, which ultimately results in a lower left ventricular end-diastolic volume, and thus, a lower preload.

#### Afterload

Unlike left ventricular preload, left ventricular afterload is directly affected by cardiopulmonary interactions. The left ventricle lies within the thoracic cavity, while most of the aorta and downstream arteries are extra-thoracic. The increase in ITP decreases the transmural pressure of the left ventricle, thereby increasing the pressure gradient between the left ventricle and the downstream extrathoracic aorta, resulting in a lower left ventricle afterload [1, 3, 5, 6, 29].

### Alpha (*α*)

Alpha displayed a relatively strong relationship to *V*_*frc*_ and SV. It is possible, in addition to compensating for the exaggerated effects of ITP on the venous blood pool, *α* also makes up for other physiological phenomena occurring during cardiopulmonary interactions which the original model is not able to account for. The most obvious being the effect of lung expansion on the CVS. As *V*_*frc*_ increases and the lungs expand, the blood vessels within the lungs are both stretched and compressed, resulting in higher pulmonary vascular resistance (PVR) [4, 6, 19, 23]. The increased PVR results in a decreased flow through the pulmonary circulation, reducing blood flow to the left ventricle and ultimately resulting in the observed lower SV. Since the model does not incorporate the right circulation to preserve identifiability [7, 25, 26], it cannot directly replicate this effect.

Another likely explanation is that, since *α* tends to decrease as PEEP is increased throughout the RM, it should be expected *α* decreases as *V*_*frc*_ is increased, because an increase in PEEP results in an increase in *V*_*frc*_, and vice versa. In the original cardiopulmonary model without *α*, errors would rise with PEEP [17, 18], as applied ITP caused modelled vena cava pressure to rise to greater values than its measured equivalent. Since *α* modulates the percentage of ITP applied to intrathoracic chambers, when PEEP is increased, *α* decreases, to compensate for the increasingly exaggerated effect ITP has on the intrathoracic chambers. Furthermore, as PEEP is increased, SV tends to diminish [2, 6, 18, 30]. Thus, it could be mathematically expected to see *α* increase with SV, since larger *α* values are associated with lower PEEP.

Counter-intuitively, *α* did not seem to show a very strong relationship to preload, where end-diastolic left ventricular volume was used as a measure for preload. Since preload is strongly linked to SV, it would be expected *α* should also display a stronger relationships than observed. This outcome is potentially due to the high noise present in the left ventricular volume measurements, potentially providing inaccurate values for the maximum volume values. Furthermore, while SV across all pigs is fairly consistent, end diastolic volume was far more pig-specific, further creating variability in the trend.

Moreover, *α* did not show a strong relationship to afterload. As stated previously, during MV, increased ITP generally works to aid left ventricular ejection. However, the majority of effects caused by cardiopulmonary interactions work to decrease SV by decreasing left ventricular preload. Since left ventricular preload and SV decrease significantly, a decrease in afterload would be difficult to observe, since left ventricular end systolic pressure decreases with lower preload. Furthermore, the output resistance was calculated using the pressure gradient between left ventricular and aortic pressure during systole. It was often observed that left ventricular pressure never rose above aortic pressure during systole in the measurements. These cases were discarded and not used for calculations. However, it is probable that the precision of the measurements may have affected the resulting calculated output resistances.

### Model comparisons

As shown in Figs 3–6, identified model outputs from the *α* model tend to closely resemble those from the original 3 chamber model. Furthermore, as shown in Fig 7, the alpha model errors closely resembled the 3 chamber model’s, while the cardiopulmonary had much higher errors than both over all PEEP steps. Due to the ITP of the cardiopulmonary model causing exaggerated vena cava and left ventricle diastolic pressures, the model has to adapt to compensate for this effect, likely leading to identified parameters which may be less precise than those of the 3 chamber model. The alpha parameter prevents this from occurring, and thus identified parameters of the *α* model more closely resemble those of the original 3 chamber model.

It is probable *α* acts as a weighting function between the cardiopulmonary model, and the original three chamber model without cardiopulmonary interactions [7, 9]. By reducing applied ITP, the *α* parameter reduces cardiopulmonary interactions to the point they no longer cause large disturbances in vena cava measurements, and thus reduce the higher model errors of the cardiopulmonary model caused by increasing PEEP. This reduction of applied ITP also causes identified model parameters to move towards those produced by the three chamber model.

Model-based care in the ICU is a highly promising prospect, allowing for far greater insights into patient conditions than is currently offered by the existing systems. The use of a combined cardiopulmonary model has the potential to act as a key diagnostic tool for both cardiovascular and pulmonary care, allowing for conjoint optimisation of both a patient’s cardiovascular and pulmonary state. Given the alpha model’s improved reliability and accuracy in high PEEP conditions, it would make a much better candidate for a bedside assistive tool than the original cardiopulmonary model. However, since identified parameters of the 3 chamber and *α* model are near identical, it could be argued incorporating the respiratory model does not provide a large enough difference in identified parameters to justify the additional model complexity. However, without the addition of the pulmonary model, it would not be possible to incorporate cardiopulmonary interactions, and thus, potentially clinically important metrics, such as stroke volume variation (SVV), would not be available from model outputs. Furthermore, the alpha model allows for potential conjoint model-based care of the pulmonary and cardiovascular states. The 3 chamber model does not allow for conjoint optimisation, and pulmonary and cardiovascular care would be performed independently. The *α* model allows for some measure of cardiopulmonary interactions while also maintaining accuracy of identified outputs. However, it is unclear as to how accurate or reliable measures such as SVV would be from the resulting model outputs.

### Limitations

A clear limitation of the paper was the small sample size used in the experiment. Data from only 4 pigs was used. A larger sample size may have yielded clearer results in the analysis and validation of *α*. However, despite the small sample size, the porcine data used in this study provided accurate information to run and validate the model.

A further limitation of the model was the physiologically incorrect application of ITP on the venous chamber. As the venous system is treated as a single lumped parameter chamber, Equation 24 states ITP is applied to the entire venous system. However, ITP would only directly influence the component of the venous system contained inside the thoracic chamber. While this presents a physiological limitation of the model, the additional invasive measurements required to separate the venous system into intra- and extra thoracic chambers, and the potential practical identifiability issues associated with identifying the parameters of an additional chamber, justify the proposed approach. As a result, ITP was applied to the entire venous blood volume, which caused exaggerated resulting cardiopulmonary effects. Therefore, although the use of the additional alpha parameter is not entirely physiologically consistent, it was able to compensate for these exaggerated effects, ultimately allowing the model to produce reliable outputs.

Finally, the model could also be improved by expanding the subset of parameters used, with only 4 of the 7 parameters identified in the optimisation process. This subset was necessary to ensure practical identifiability of the model from the clinically feasible measurements available, as the additional complexity of the model resulted in the exclusion of both the input resistance and venous elastance parameters from optimisation. Despite this, the subset proved sufficient, as the model was able to accurately reproduce system dynamics with low model errors.

## Conclusions

The original cardiopulmonary model, while having the ability to incorporate cardiopulmonary interactions, resulted in increased model errors at high PEEP. The addition of *α* proved to be a promising alternative, reducing model errors at high PEEP. While *α* may display fairly convincing relationships to physiological measures such as *V*_*frc*_ and SV, these relationships can be explained through *α*’s inverse relationship to PEEP. Furthermore, *α* seems to act more as a weighting function between the cardiopulmonary model and the original three chamber CVS model, reducing cardiopulmonary interactions in order to maintain a stable model error.

Identified parameters from the *α* model closer resemble those of the original 3 chamber model, rather than those from the cardiopulmonary model, especially under high PEEP conditions. This is largely due to the high errors seen in the cardiopulmonary at high PEEP levels, caused by the exaggerated effects of ITP. Ultimately, *α* works to reduce these effects, thereby bringing model outputs and identified parameters closer to those of the original 3 chamber model.

Ultimately, the *α* model has shown to be more stable than the original cardiopulmonary model, making it a good candidate for model based care. Even though it produces similar identified parameters to the 3 chamber model, it is also able to produce cardiopulmonary interactions in model outputs, providing potentially useful clinical metrics, such as stoke volume variation (SVV). These clinical metrics can act as key indicators for critical procedures, such as fluid therapy, and may ultimately improve survival probability for patients in critical condition. However, it is uncertain how reliable or accurate these measures are given the diminished nature of the cardiopulmonary interactions produced in the *α* model. Consequently, it is uncertain whether cardiopulmonary interactions of the *α* model justify its additional complexity, especially when considering the low availability of clinical data in an ICU setting.

The model clearly has the ability to provide key insights into a patients cardiopulmonary state, and to be used as a potentially lifesaving bedside assistive tool. However, more work is required to better understand and model the complex relationships between the cardiovascular and respiratory systems.
